# Methods and system for recording human physiological signals from implantable leads during spinal cord stimulation

**DOI:** 10.3389/fpain.2023.1072786

**Published:** 2023-03-03

**Authors:** Ahmed Ramadan, Seth D. König, Mingming Zhang, Erika K. Ross, Alexander Herman, Theoden I. Netoff, David P. Darrow

**Affiliations:** ^1^ Department of Biomedical Engineering, University of Minnesota, Minneapolis, MN, United States; ^2^ Department of Neurosurgery, University of Minnesota, Minneapolis, MN, United States; ^3^ Department of Psychiatry and Behavioral Sciences, University of Minnesota, Minneapolis, MN, United States; ^4^ Clinical and Applied Research, Abbott Neuromodulation, Plano, TX, United States

**Keywords:** epidural spinal cord stimulation, evoked compound potential, neuromodulation, stimulation artifact removal, neural signal processing

## Abstract

**Objectives:**

This article presents a method–including hardware configuration, sampling rate, filtering settings, and other data analysis techniques–to measure evoked compound action potentials (ECAPs) during spinal cord stimulation (SCS) in humans with externalized percutaneous electrodes. The goal is to provide a robust and standardized protocol for measuring ECAPs on the non-stimulation contacts and to demonstrate how measured signals depend on hardware and processing decisions.

**Methods:**

Two participants were implanted with percutaneous leads for the treatment of chronic pain with externalized leads during a trial period for stimulation and recording. The leads were connected to a Neuralynx ATLAS system allowing us to simultaneously stimulate and record through selected electrodes. We examined different hardware settings, such as online filters and sampling rate, as well as processing techniques, such as stimulation artifact removal and offline filters, and measured the effects on the ECAPs metrics: the first negative peak (N1) time and peak-valley amplitude.

**Results:**

For accurate measurements of ECAPs, the hardware sampling rate should be least at 8 kHz and should use a high pass filter with a low cutoff frequency, such as 0.1 Hz, to eliminate baseline drift and saturation (railing). Stimulation artifact removal can use a double exponential or a second-order polynomial. The polynomial fit is 6.4 times faster on average in computation time than the double exponential, while the resulting ECAPs’ N1 time and peak-valley amplitude are similar between the two. If the baseline raw measurement drifts with stimulation, a median filter with a 100-ms window or a high pass filter with an 80-Hz cutoff frequency preserves the ECAPs.

**Conclusions:**

This work is the first comprehensive analysis of hardware and processing variations on the observed ECAPs from SCS leads. It sets recommendations to properly record and process ECAPs from the non-stimulation contacts on the implantable leads.

## Highlights

1. Evoked compound action potentials (ECAPs) can provide objective measurements of the spinal cord's response to electrical stimulation, but they are partially obscured by stimulation artifacts. In this study, we test stimulation artifact removal by fitting a curve to the raw recorded potentials using a novel second-order polynomial, which is then subtracted to reveal the clean ECAPs. This method was as accurate as a double exponential and significantly faster in computation, thus better suited to real-time closed-loop applications.
2. Our results indicate that a 100-ms median filter or an 80-Hz high pass filter preserved the ECAPs' shape for offline analysis, but a 3-kHz low pass filter significantly altered the shape. This study's exploration of filter settings provides suggested signal processing steps suited to an embedded stim-and-record system.
3. A minimum of 8-kHz sampling rate is needed for accurate ECAP metrics (peak-valley amplitude and the first negative peak time). Down-sampling the 32-kHz data to 8 kHz resulted in similar metrics, but further down-sampling to 4 kHz or less significantly affected the quality of the data. It suggests that an 8-kHz sampling rate would be necessary on an embedded stim-and-record system.
4. A common method of stimulation artifact removal is to switch between anodic and cathodic stimulation and then sum the resulting responses under the hypothesis that the stimulation artifact reverses but the neural response does not. However, in this study, the evoked responses off the cathodic and anodic stimulation had different delays and amplitudes. Therefore, we suggest not using the switching average to remove the stimulation artifact if the goal is to accurately measure ECAP properties.

## 1. Introduction

Stimulation of the spinal cord has long been used to effectively treat lower back pain (1) and complex regional pain syndrome (CRPS) (2). One of the challenges faced during spinal cord stimulation (SCS) therapy is loss of chronic efficacy of the treatment (3). Underlying reasons for this change of therapy may include device failure, lead migration, habituation of SCS treatment, etc (3–5). Finding a control signal that can provide necessary notification or even adjustment to treatment is necessary for long-term SCS therapy and user experience improvement. One potential biomarker could be the evoked compound action potentials (ECAPs) generated during SCS. The ECAP signal represents the activation of neuronal fibers upon delivery of electrical stimulation to the dorsal column fibers in the spinal cord (6). Recently, some research work also indicated that the recorded evoked responses from the non-stimulation contacts during SCS could contain electromyography (EMG) signals from nearby muscle contractions as well as ECAPs (7). Therefore, the epidural spinal recordings (ESR) are collected from electrodes placed in the epidural space and contain multi-modality signal components such as the ECAP, the evoked muscle response, stimulation artifacts, and cardiac response (8).

Adjusting stimulation parameters based on the change of the evoked responses may influence the therapeutic outcome (9, 10). However, there is insufficient understanding of how the evoked responses, such as ECAP signals or the evoked EMGs during spinal cord stimulation, are connected to the therapy outcome. In addition, the stimulation artifact could severely contaminate the evoked responses and it can be easily included in the quantification of the signals (11). As a result, command signals generated by simple quantification of such evoked responses can contain different physiological signals and can be contaminated by stimulation artifact. Thus, using it to adjust stimulation parameters could inaccurately adjust the therapy and sometimes even mislead the SCS therapy.

To study ECAP signals and its potential implementation for closed-loop SCS, a good methodology needs to be established to ensure reliable recordings and appropriate quantification of the responses collected from the SCS leads. In this study, we will explore these topics from the following three aspects: (1) recommendation of the settings for data acquisition systems, (2) impact of different curve fittings for stimulation artifact removal and their computational cost, and (3) effects of different filtering options on detected ECAP properties. While ECAPs have been extracted in real time and used for closed-loop control of stimulation settings (10, 12), the hardware configuration and algorithms used are trade secrets. Therefore, our goal here is to create a public and common methodology for measuring ECAPs to be used by the general scientific community.

## 2. Materials and equipment

### 2.1. Stim/record setup

ECAPs have been measured using many different devices and have been reported in the literature with many different recording and filtering protocols. Table 1 summarizes many of the pertinent technical details used to record ECAPs in previous studies including the devices used to stimulate and record ECAPs in the spinal cord of humans to treat chronic pain.

**Table 1:** A summary of the devices and settings used to record ECAPS in the human spine.

Human study	fs	Lead configuration	Waveform Generator/ Stimulator/Amplifier/ Digitizer (G/S/A/D)	Stim electrodes/stim waveform	Online/offline filter	Reference electrode	Industry partner
(13)	24.4 kHz	two 8-contact leads formed a linear 16-channel array	WPI A385 current source^G,S^/TDT RZ5 amplifier and bioprocessor system^A,D^	guarded cathode (3 channels)/symmetric biphasic	7.5 kHz antialiasing LP/3 kHz LP	most rostral electrode	–
(14)	30 kHz^a^	two 8-contact leads with an overlap of 2–4 contacts	SM Multi-Channel System MkII)^G,S,A^/UEI DAQ^D^a^^	tripolar^b^/symmetric biphasic	NA^c^	NA^c^	Saluda Medical
(15, 16)	NA^c^	two staggered 8-contact leads	NI hardware^G^a^^/Digitimer DS5^S^/Digitimer D440^A^/NI hardware^D^a^^	guarded cathode/ symmetric biphasic	NA^c^	differential of adjacent electrodes on a single lead	Medtronic
(9)	NA^c^	two staggered 8-contact leads	NI hardware^G^a^^/Digitimer DS5^S^/Digitimer D440^A^/Biopac MP160^D^	guarded cathode/symmetric biphasic	NA^c^	differential of adjacent electrodes on a single lead	Medtronic
This study	32 kHz	two 8-contact leads with an offset of 0–2 contacts	MATLAB^G^/Neuralynx ATLAS system^S,A,D^	bipolar/asymmetric biphasic	8 kHz antialiasing LP, 0.1 Hz HP/100 ms Med	most rostral electrode on the non-stimulating lead	This study

LP, low pass filter; HP, high pass filter; Med, median filter; SM, Saluda Medical; UEI, United Electronic Industries; NI, National Instruments; fs, sampling frequency.

^a^ From a referenced article within the mother article.

^b^ Tripolar is supposedly the same as guarded cathode.

^c^ Not available in the article's text.

**Figure 1:**
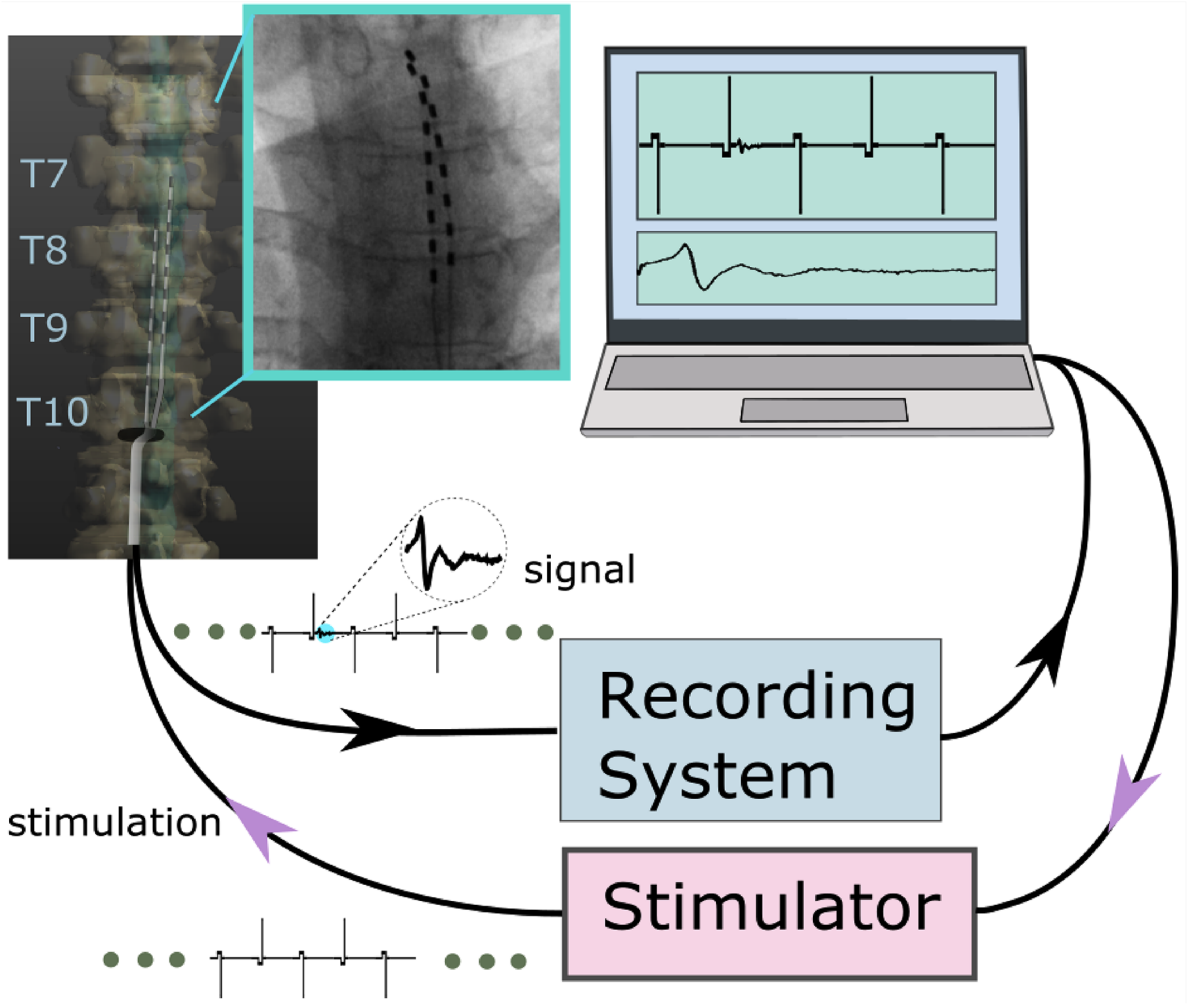
An overview of the stim-record system (ATLAS) connected to the externalized epidural leads implanted in a participant. The ATLAS Stim headbox delivered biphasic interleaved waveforms to two electrodes. The recording system recorded from all 16 channels.

**Figure 2:**
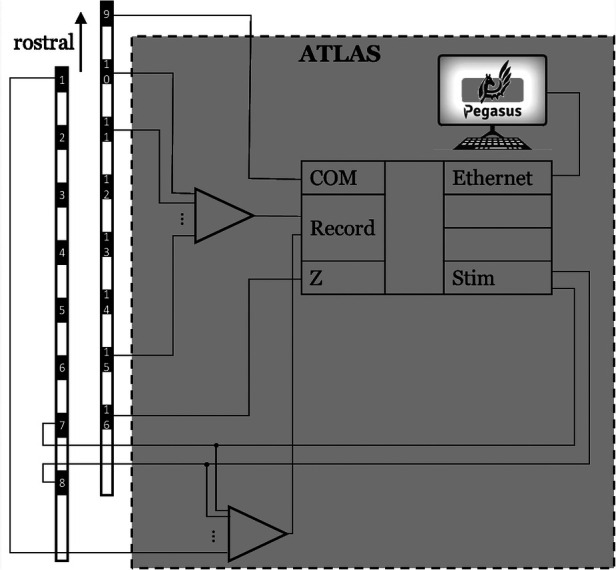
The hardware wiring featured an active ground input COM and an active ground output Z (in a hardware update, COM and Z were renamed REF and GND, respectively). The ATLAS system controlled what electrodes to be stimulated (7 and 8 in this demonstration).

In this study we use an ATLAS neurophysiology system (Neuralynx, Bozeman, Montana, United States) to simultaneously stimulate and record through percutaneous electrodes implanted in the spinal column (Figures 1, 2). The ATLAS Stim headbox is an investigational device that provides a unity gain buffer to the recordings and delivers user-defined stimulation waveforms at 40 kHz for up to 10 s. The system's input was digitized at 24 bits over a range of ±132 mV at 32 kHz and transmitted to a PC (ATLAS workstation) *via* a fiber optic ethernet cable. The Pegasus software controls online filters, user-defined measurement range, reference selection, and real-time signal display. It also saves the data in two formats: a continuously sampled format (.ncs) and raw data format (.nrd). The former has the data in 16-bit resolution, user-defined measurement range, and filtered if any online filter was enabled. The latter has the raw data in 24-bit resolution, the full ±132 mV range, and no filters. In this paper, we used the continuously sampled data with a user-defined range of ±100 mV.

An advantage of the ATLAS Stim headbox is that it allows direct stimulation to any electrode without disconnecting it from the recording amplifier, which dramatically speeds up the time needed to record from many different stimulation sites and reduces artifacts caused by connecting and disconnecting electrodes between recording and stimulation headboxes. The ATLAS system also can rapidly recover, in as little as 200 µs if the recorded signal saturates the ±132 mV range during stimulation. The ATLAS Stim headbox can deliver any arbitrary waveform designed by the user that meets several basic safety design requirements. The stimulation waveform can be generated by any software package and stored as a “.wav” file where the amplitude is defined within [−1, 1] and the final amplitude of the stimulation to be delivered to the electrodes can be scaled using the software interface. While this device was used for this study, we believe many of the lessons learned here will generalize to other devices.

**Figure 3:**
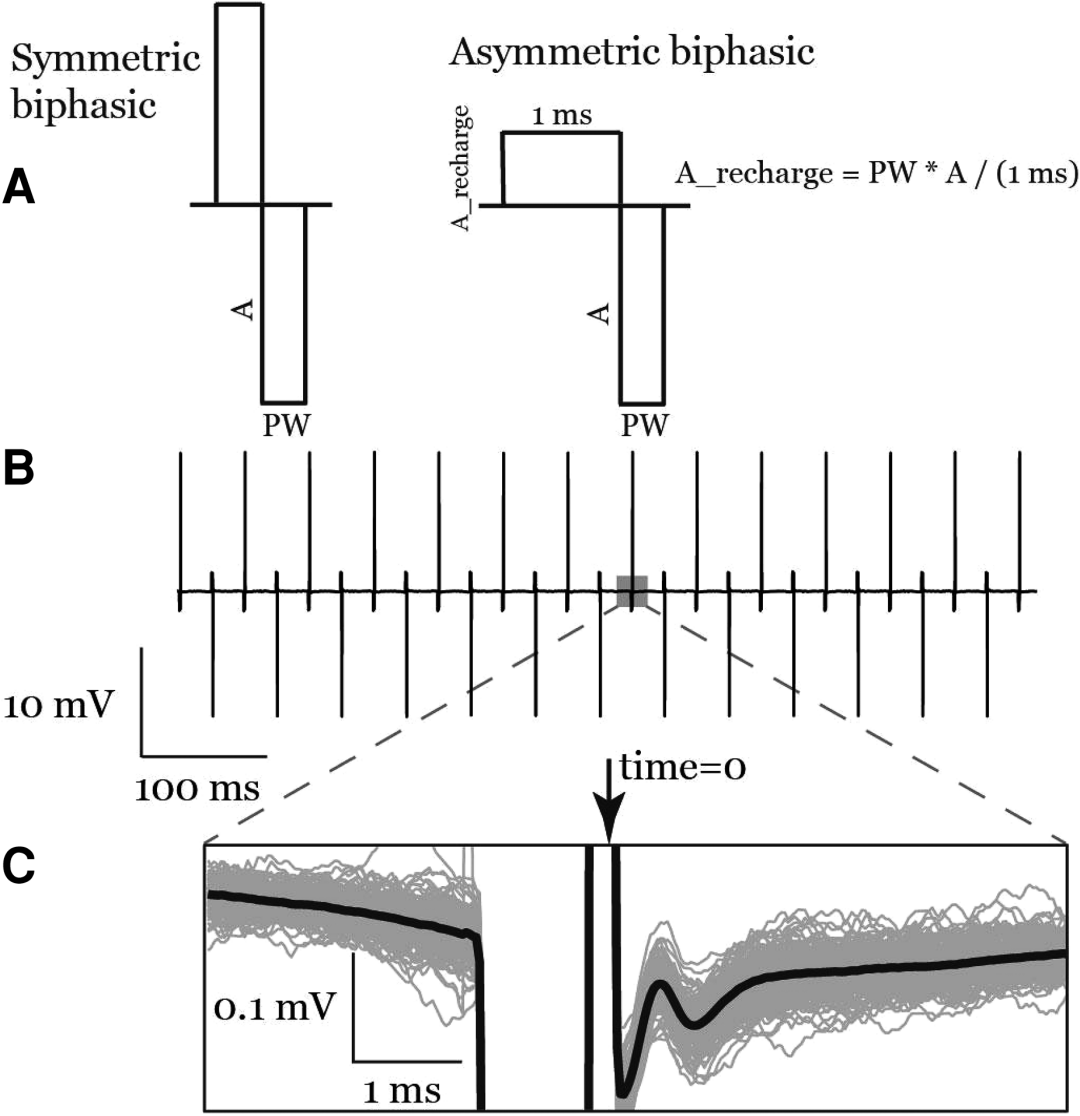
(**A**) the symmetric biphasic and asymmetric biphasic (used in this study) stimulation waveforms. (**B**) A raw recording of a train of asymmetric biphasic interleaved pulses. (**C**) Inset shows anodic pulse ECAPs signal in the average waveform (bold line) as well as the individual traces (gray lines).

Stimulation waveforms for our experiments were programmed using a MATLAB (R2021b, Mathworks, Natick, Massachusetts, United States) script and stored as “.wav” files. To minimize the artifact in our recorded signal after the stimulation, we used an asymmetric biphasic charge-balanced waveform, as shown in Figure 3, with a 1-ms charge balance phase followed by the stimulation pulse. We also designed stimulation waveforms to switch the polarity every pulse (Figure 3). Here we define the polarity of a pulse as anodic or cathodic according to the polarity of the second phase on the recording electrode, where anodic polarity is positive (17). Stimulation was delivered through the most caudal channels of the two implanted leads to maximize the distance between stimulation and recording electrodes.

## 3. Methods

### 3.1. Participants

Two participants with chronic pain trialing percutaneous electrodes were recruited for this study. Demographics of the patients are listed in Table 2. The study protocol was approved by the University of Minnesota's IRB (#STUDY00013100) and was registered on ClinicalTrials.gov (#NCT04938245). All participants signed an informed consent to participate. Participants had 1 to 3 follow-up visits, depending on each participant's availability, in the week following implantation during the therapy trial period while electrode leads were externalized. Participants were not anesthetized during the experiment.

**Table 2:** Participants’ demographics

ID	Sex	Age	Pain region	electrode placement	height/weight
1	M	56	right leg and back	T8-T10. Left side is lower than right by two electrodes	170 cm/73 kg
2	M	53	Abdominal [Complex regional pain syndrome (CRPS)]	T6-T8. Left side is lower than right by one electrode	

### 3.2. Surgery

Abbott's Percutaneous Octrode™ leads were implanted under fluoroscopy. The participants were placed prone on the operating table under MAC. Local anesthetic was injected along two parallel trajectories towards the interspinous space. Tuohy needles were advanced past the spinous process of L2 towards the inferior portion of the lamina of L1. Once sufficiently close to the inferior edge of the lamina, the stylet was removed, and a loss-of-resistance syringe was attached to the Tuohy needle. Millimeter advancements were made while testing for loss of resistance upon entry to the epidural space. Once achieved, the Octrode™ lead with stylet was passed through the needle cephalad under fluoroscopy and advanced carefully to the T7/8 disk space. Similarly, the same procedure was performed on the contralateral side, resulting in parallel percutaneous electrodes. The leads were offset by two contacts with the right side more cephalad.

### 3.3. Hardware settings

#### 3.3.1. Sampling frequency

To test the effect of the sampling frequency on the ECAP morphology, we down-sampled our data from 32 kHz to 8, 4, and 2 kHz using MATLAB's *decimate* function with a finite impulse response (FIR) antialiasing filter using 30 taps and a cutoff at Nyquist frequency before down-sampling.

#### 3.3.2. Online filter

We collected data from the first participant without any online filters enabled in the ATLAS system. As a result, some recordings saturated the hardware amplifier. After saturation, we found that the signals continued to be distorted as if some high pass hardware filter had been added. After the first participant, we enabled a 0.1 Hz pre-emphasis (high pass) filter, which prevented amplifier saturation in subsequent experiments. Note, the raw data (24-bit, .nrd) was never saturated.

#### 3.3.3. Referencing

The ground electrode was a patch on the back in participant #1 and an on-lead electrode (#16) in participant #2. The on-lead reference electrode was #9 to be as far as possible from the stimulation electrodes (7 & 8), see Figure 2.

#### 3.3.4. Lead positions

Since the participants had leads implanted as a medical therapy, their neurosurgeons had full control on lead positioning. The two participants had offsets by 1 to 2 channels between the two leads.

### 3.4. Data processing

Continuous data (16-bit, .ncs) was imported and basic signal processing performed in MATLAB using the Fieldtrip toolbox (18). Most channels close to stimulation electrodes (up to 2 channels away) were highly corrupted by the stimulation artifact. ECAPs were measured on the remaining electrodes. We chose to report ECAPs from channel 12 across the patients for the analysis purposes here (see Figure 2 for channel numbers on each lead).

#### 3.4.1. Offline filter

To determine the effects of filtering on ECAP detection and characterization, three common filtering schemes were applied: (1) a median filter of a 100-ms window using MATLAB's *medfilt1*; (2) a high pass filter with an 80-Hz cutoff frequency (4th order Butterworth applied in forward and reverse) using MATLAB's *butter* and *filtfilt* functions; (3) a low pass filter with 3-kHz cutoff frequency [50-tap finite impulse response (FIR) filter] using MATLAB's *fir1* and signal padding to account for the linear phase shift to closely replicate filter settings used in (19). Not all papers shown in Table 1 reported their filter settings. To compare our results with theirs, assuming that these papers did in fact not apply any software post-process filtering, we show the effects of no filtering on the ECAP waveform. We also characterized the filters themselves and show their impulse response, to disambiguate the effects of the ringing occurring from filtered stimulation artifact from the ECAP.

#### 3.4.2. Stimulation time

Time zero for each pulse was defined as the time of the largest artifact derivative on the trailing edge of the pulse (Figure 3). To estimate the stimulation times: first, the signal was rectified, to generalize the method for anodic and cathodic pulses; second, the derivative was calculated by taking the sample difference; last, the largest negative deflection with an amplitude greater than or equal 30% of the maximum was selected using MATLAB's *findpeaks* function with a minimum exclusion window of 50 samples between detected stimulation times.

The peri-stimulation aligned responses were then baseline corrected by subtracting the average of the traces in the time window 2–5 msec preceding the stimulation time. Preliminary analysis showed that there was a time offset between anodic stimulation pulse induced ECAPs (anodic ECAP) and cathodic stimulation pulse induced ECAP (cathodic ECAP), as well as different ECAP amplitudes. Thus, in this study, we will separately analyze ECAPs triggered by different stimulation polarities.

#### 3.4.3. Artifact removal

The stimulation artifact on the recording electrodes was most often orders of magnitude larger than the evoked response, and the stimulation artifact decay often lasted longer than the ECAP response depending on the distance from the recording electrode to the stimulation electrodes. Therefore, it is necessary to remove the stimulation artifact to accurately quantify the evoked response. A goal is to fit a low enough order model to capture the recovery from the stim artifact but to leave the ECAP properties unaffected after subtraction. To remove the stimulation artifact, we fit a curve to the data measured in a time window between 0.375–4 ms following the stimulation time and then subtracted out the subsequent curve. Stimulation artifacts were fitted with a single exponential (exp1), a double exponential (exp2), and a 2nd order polynomial (poly2). In each case, the best-fit curve was subtracted from the average data to obtain the ECAP. We used MATLAB's *fit* function with its default optimization options. For exp1 and exp2, nonlinear least squares problems were solved with a trust-region algorithm. In poly2, linear least squares problems were solved with a QR factorization algorithm. The computation time for each function fit was measured using MATLAB's *timeit* function on a PC with an Intel Core i7–5820 K CPU.

#### 3.4.4. ECAP metrics & peak detection

To quantify the effect of different settings on ECAP morphology, peak-valley amplitude (P2-N1) and N1 time will be measured. First, the N1 valley was determined using MATLAB's *findpeaks* function on the ECAP signal from 0.375 ms to 2.1875 ms with a minimum peak height of 0.1 µV. Then, the P2 peak was found in the window from the N1 location to 2.1875 ms with a minimum peak height of 0.1 µV. We do not report metrics based on the P1 peak since it was not observed in all cases as it occurred earlier than the start of our analysis window.

#### 3.4.5. Statistics

Linear models using MATLAB's *fitlm* were used to compare the different settings (e.g., filtering) on common ECAP metrics (N1 time and peak-valley amplitude). An example model is “N1_time∼group”, where the categorical variable “group” takes the levels: “exp2”, “exp1”, and “poly2”. 20 observations were included in the analysis as listed in Table 3. An observation is one or more concatenated trials of the same stimulation parameters per participant per visit. A trial is a 10-s recording block during stimulation with fixed parameters. The different processing settings (e.g., filtering) were categorical independent variables. Since we had 16 models in this analysis, we used Bonferroni correction and set the significance to *p* < 0.0031 (0.05/16).

**Table 3:** The 20 observations across participants and stimulation parameters

# of observations	participant	visit	stim amplitude (mA)	stim pulse width (us)	stim frequency (Hz)	stim/measurement electrodes
4	2	1	3.5, 4, 4.5, 5	150	38	7,8/12
7	1	2	5, 5.6, 6, 6.5, 7, 7.5, 8	150	38	7,8/12
5	1	3	5, 5.6, 6, 6.5, 7	150	38	7,8/12
4	1	3	3, 3.5, 4, 4.5	350	38	7,8/12

## 4. Results

### 4.1. Processing settings

#### 4.1.1. Artifact removal

**Figure 4:**
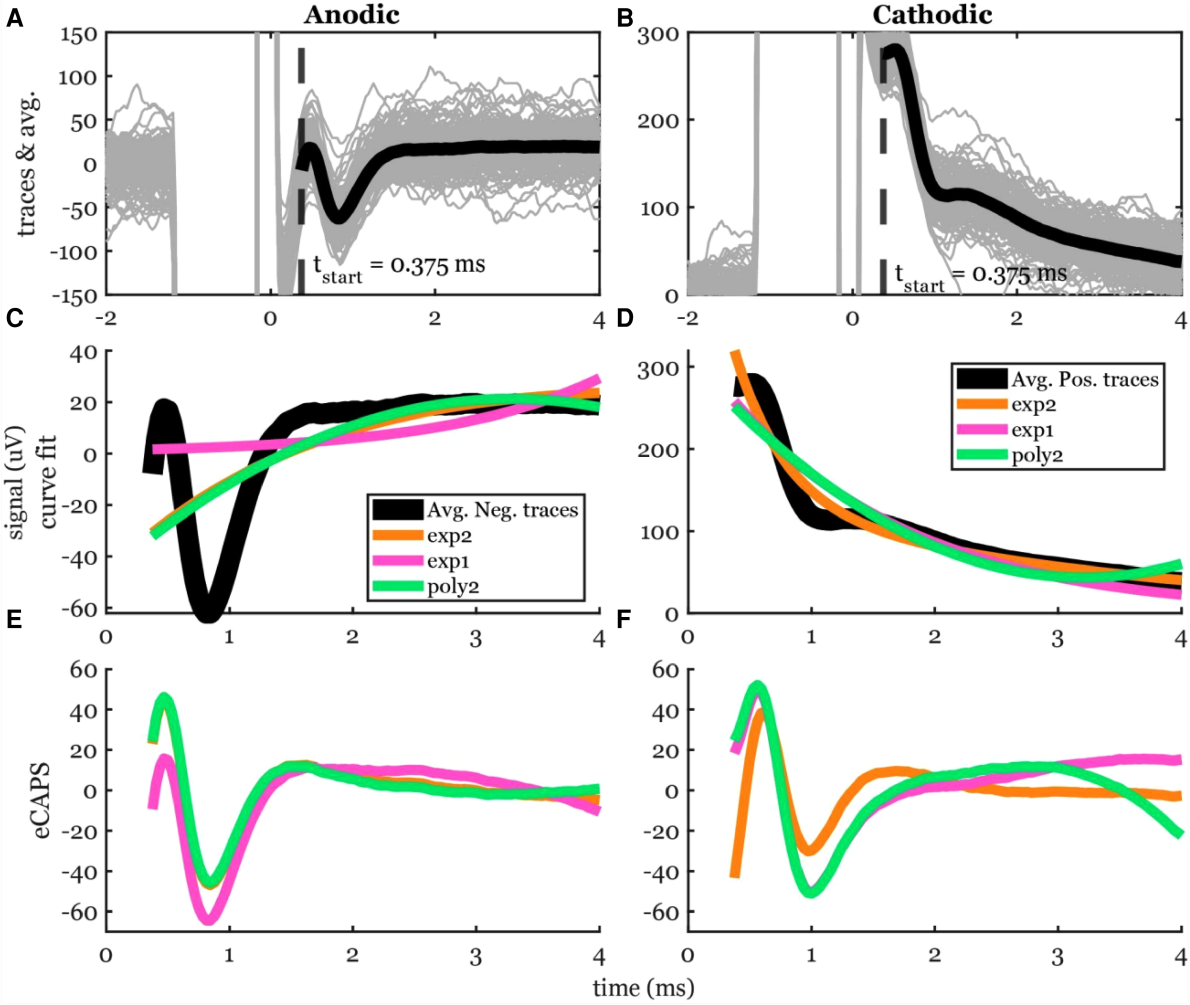
Stimulation artifact removal steps demonstrated in a 5-mA stimulation of participant #2. (**A,B**) Raw traces (gray lines) filtered with the median filter grouped into cathodic and anodic classes and averaged (black lines) separately. (**C,D**) Three curves (exp2: double exponential, exp1: single exponential, and poly2: 2nd order polynomial) were fit to the average signal. The time window for the artifact removal algorithm was [0.375, 4] ms. (**E,F**) The resulting ECAP after subtracting the estimated stimulation artifact.

Figure 4 shows averaged data across many stimuli including both the ECAP and stim artifact separated by anodic and cathodic stimulation. Curves of different functions were fit to the original recording containing stimulation artifact and then subtracted from the original recording. The best-fit curves and the resulting ECAPS are shown in Figure 4. The artifact morphology looked different between the two stimulation polarities.

**Figure 5:**
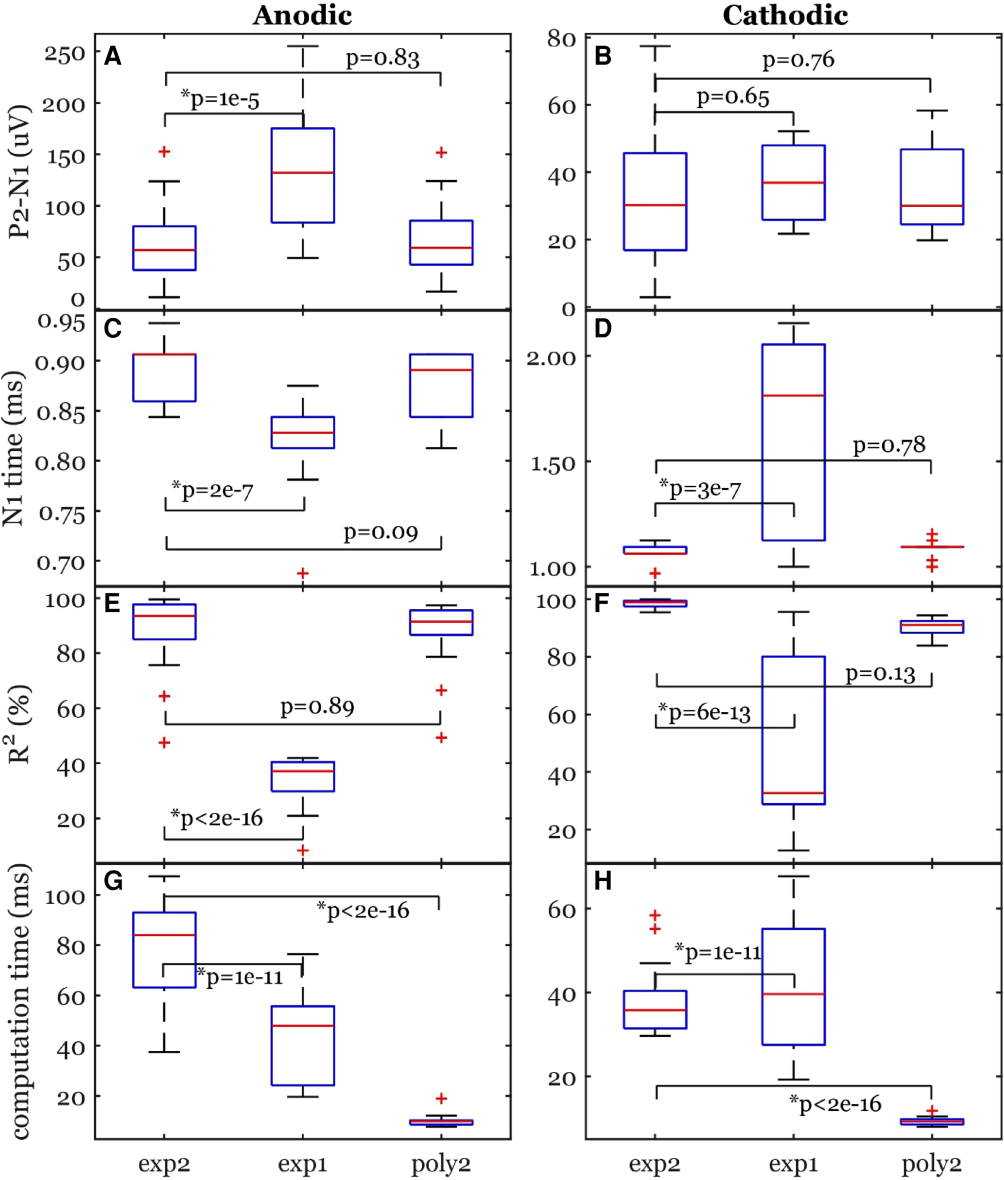
Common ECAP characteristics: (**A,B**) peak-valley amplitude (P2-N1) and (**C,D**) N1 time; (**E,F**) the goodness of fit across three curve fittings; (**G,H**) the computation time for a fit. exp2: double exponential, exp1: single exponential, poly2: 2nd order polynomial. The *p*-values come from linear models. The data included 20 observations from two participants with stimulation amplitudes sufficient to evoke ECAP in each participant.

The peak-valley amplitude (P2-N1) was affected by the function used to fit the stimulation artifact (Figure 4). In some cases, the peak-valley amplitude could not be calculated since either N1 and/or P2 were not detected. Out of the 20 observations, peak-valley amplitude was not calculated in four observations using exp1 in anodic ECAP, and 16 and 14 observations using exp1 and poly2 respectively in cathodic ECAP. Peak-valley amplitude was calculated in the remaining observations and curve fits. There was no significant difference in peak-valley amplitude between exp2 and poly2 (Figure 5). The exp1 yielded significantly different peak-valley compared to the exp2 in anodic ECAP only. The average peak-valley amplitude difference when using exp1 and poly2 compared to exp2 were −67.5 and −3.0 µV in anodic ECAP, and −10.4 and −17.3 µV in cathodic ECAP, respectively.

The N1 peak times were affected by the fit type as well (Figure 5). The algorithm could not detect N1 peaks in four cases in anodic ECAP and one case of cathodic ECAP when using exp1. The N1 times of exp2 were different from those of exp1 but not different from those of poly2 (Figure 5). The average N1-time differences of exp1 and poly2 from exp2 were 74 and 20 µs in anodic ECAP, and −528 and −25 µs in cathodic ECAP, respectively. Given that our time resolution is 31.25 µs (1/32 kHz), the average difference of N1 time in poly2 from exp2 is negligible.

Exp2 had the best fit to the stimulation artifact (R^2^ in Figure 5) resulting in the ECAP with the cleanest morphology (Figure 4). The average R^2^ improvement of exp2 over exp1 and poly2 was 54.5% and 0.5% in anodic ECAP and 49.0% and 8.1% in cathodic ECAP, respectively. For further analysis, we used the exp2 function to remove the stimulation artifacts.

The computation times for calculating the best-fit curves were significantly different between exp2 and the other two functions (Figure 5). The poly2 fit had the shortest computation time. Compared to poly2, the computation time of exp2 was 8.7 and 4.2 times longer for anodic and cathodic ECAPs respectively, while the exp1 computation time was 4.6 and 4.4 times longer for anodic and cathodic ECAPs respectively.

**Figure 6:**
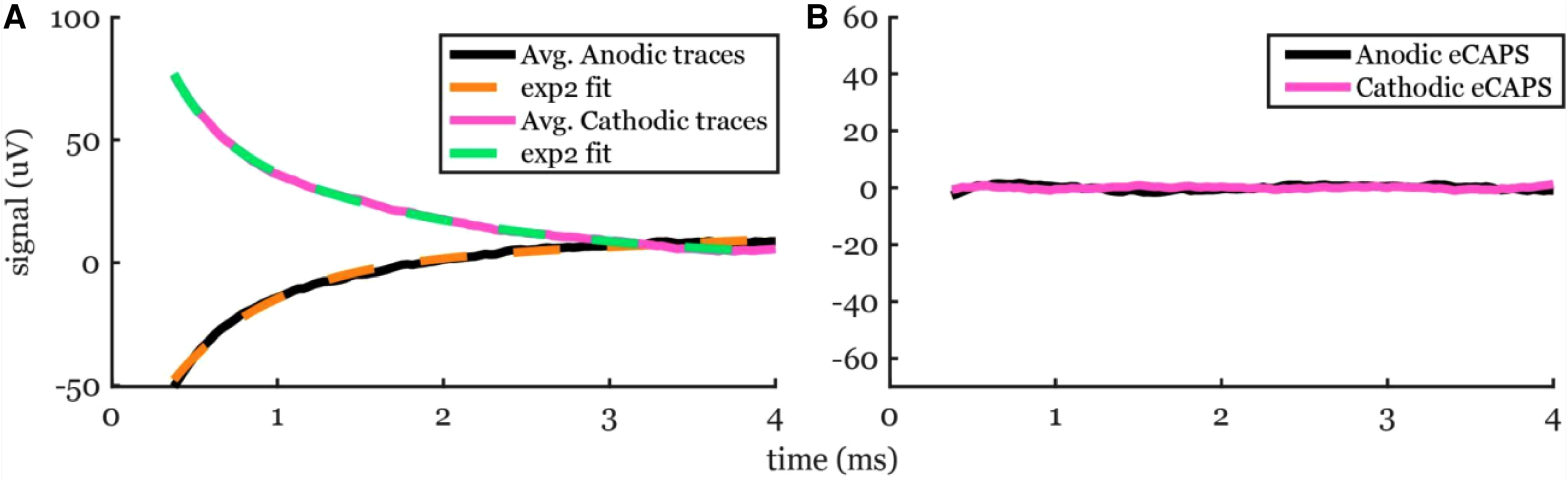
Artifact removal using exp2 fit to a recording from a subthreshold 1-mA stimulation in participant #2. (**A**) The averaged traces (solid lines) and the best-fit curves (dashed lines). (**B**) No ECAP was observed, as expected.

We also tested exp2 fitting on low stimulation amplitudes where no ECAP was observed to ensure that these methods do not artificially create detectable peaks and valleys that could mistakenly be categorized as ECAPs. An example of a stimulation without an ECAP at a subthreshold stimulation amplitude of 1 mA is shown in Figure 6. The fit was nearly perfect with R^2^ > 99% for anodic pulses and cathodic pulses and no detectable peaks or valleys were observed.

#### 4.1.2. Offline filtering

**Figure 7:**
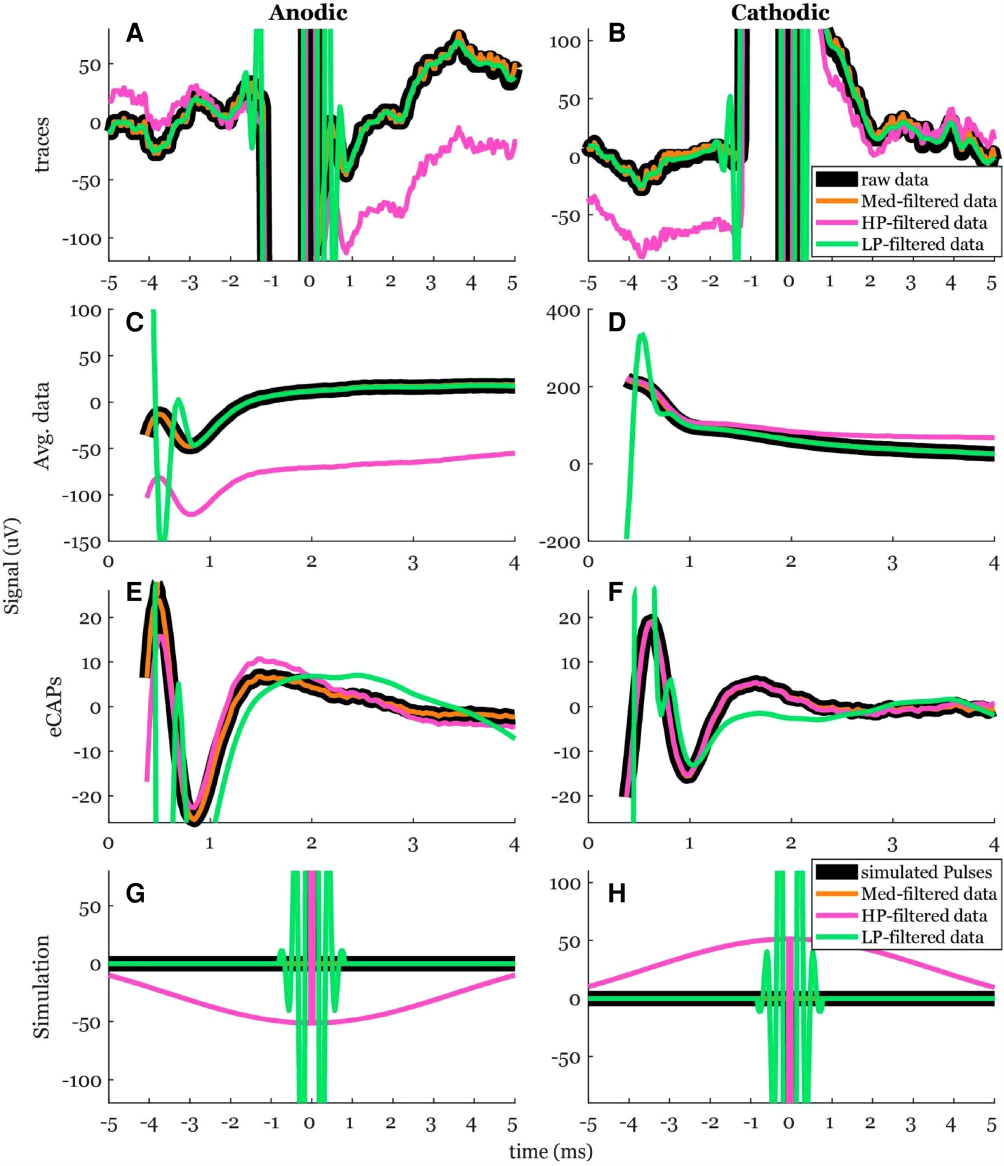
The effects of using offline digital filters: a median (Med) filter with a 100-ms window, a 4th order Butterworth high pass (HP) filter with 80-Hz cutoff frequency applied in forward and reverse, and a 50-tap FIR low pass (LP) filter with 3 kHz cutoff frequency. (**A,B**) A segment of the time-trace for anodic and cathodic ECAP. (**C,D**) The averaged data. (**E,F**) The resulting anodic and cathodic ECAP using exp2 fit. (**G,H**) The effects of each filter on an impulse function in simulation.

We tested the effects of a median filter (Med), a high pass filter (HP), a low pass filter (LP), and no filtering (raw) on the recordings (Figure 7). The HP introduced a noticeable offset to the raw data after the anodic pulse and before the cathodic pulse. In simulations, a bump-like offset is noted with the HP (Figure 7). The Med did not introduce any offsets and followed the raw recording very well. The LP introduced an observable ringing effect close to the stimulation time and again in the simulated pulses.

**Figure 8:**
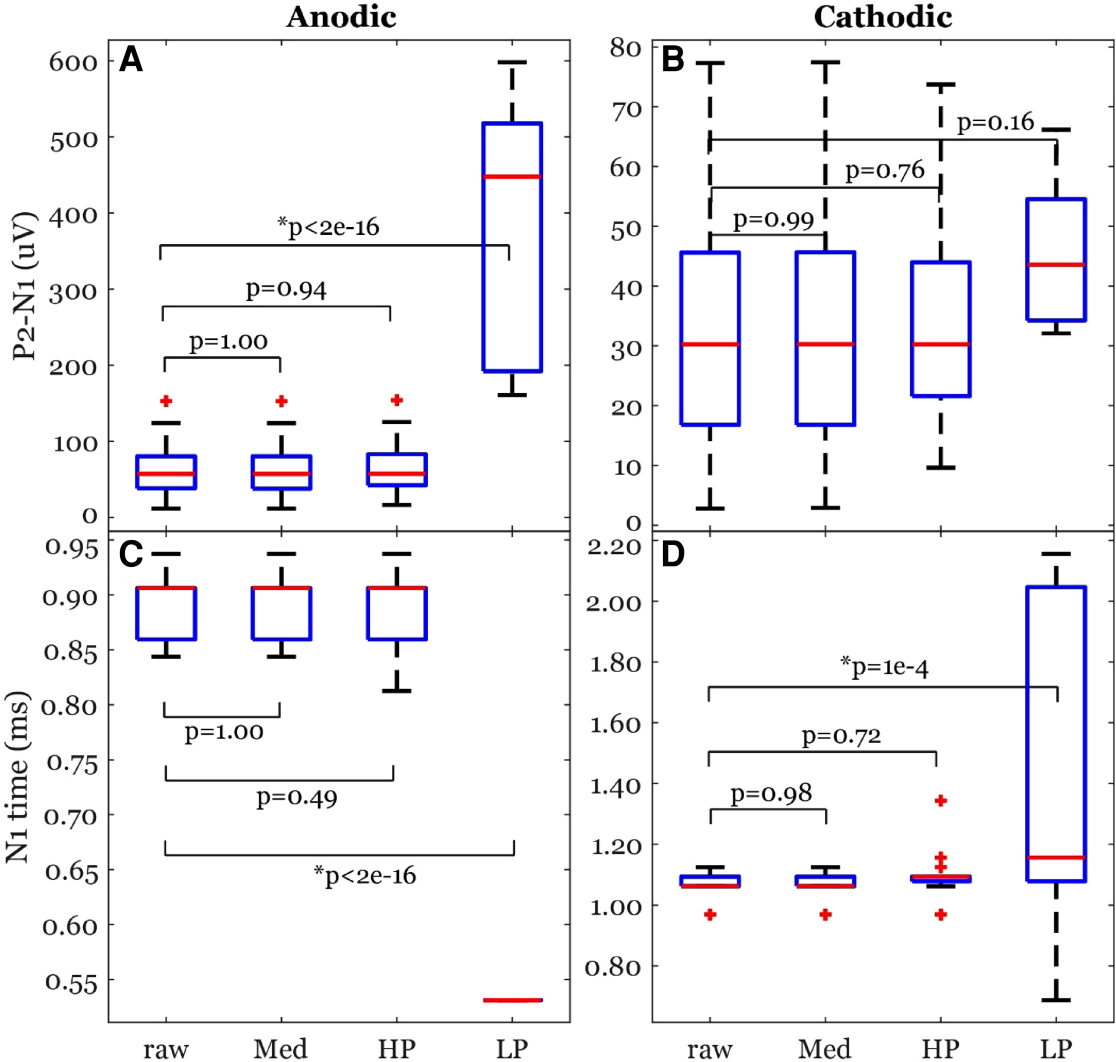
Common ECAP characteristics: (**A,B**) peak-valley amplitude (P2-N1) and (**C,D**) N1 time; across the four filtering options; raw: no filtering, Med: median filter, HP: high pass filter, and LP: low pass filter. The *p*-values come from a linear model. The data included 20 observations from two participants with stimulation amplitudes sufficient to evoke ECAP in each participant.

The peak-valley amplitude (P2-N1) was affected by the filter type (Figure 7). In some cases, the peak-valley amplitude could not be calculated since either N1 and/or P2 were not detected. Out of the 20 observations, peak-valley amplitude was not calculated in two cases of HP and 15 cases of LP in cathodic ECAP. The peak-valley amplitude was not different between raw and each of Med and HP (Figure 8). The LP peak-valley amplitude was significantly different from that of the raw recordings in anodic ECAP but not in the cathodic ECAP, which is probably affected by the small sample size of LP observations. The average peak-valley amplitude differences of Med, HP, and LP from raw were 0.1, −2.1, and −311.6 µV in anodic ECAP, and −0.0, 1.2, and −6.9 µV in cathodic ECAP, respectively.

The N1 times were affected by the filter type as well (Figure 7). The algorithm detected N1 times for all cases. The N1 times followed a similar pattern to the peak-valley amplitude with no significant difference between raw and each of Med and HP results. However, the N1 times of the LP filtered signals were significantly different from raw signal (Figure 8). The average N1-time differences of Med, HP, and LP from raw were 0, 6, and 363 µs in anodic ECAP, and 2, −28, and −320 µs in cathodic ECAP, respectively. Given that the time resolution is 31.25 µs (1/32 kHz), the averaged differences of N1-time in Med and HP from raw is technically zero.

### 4.2. Hardware settings

#### 4.2.1. Sampling frequency

**Figure 9:**
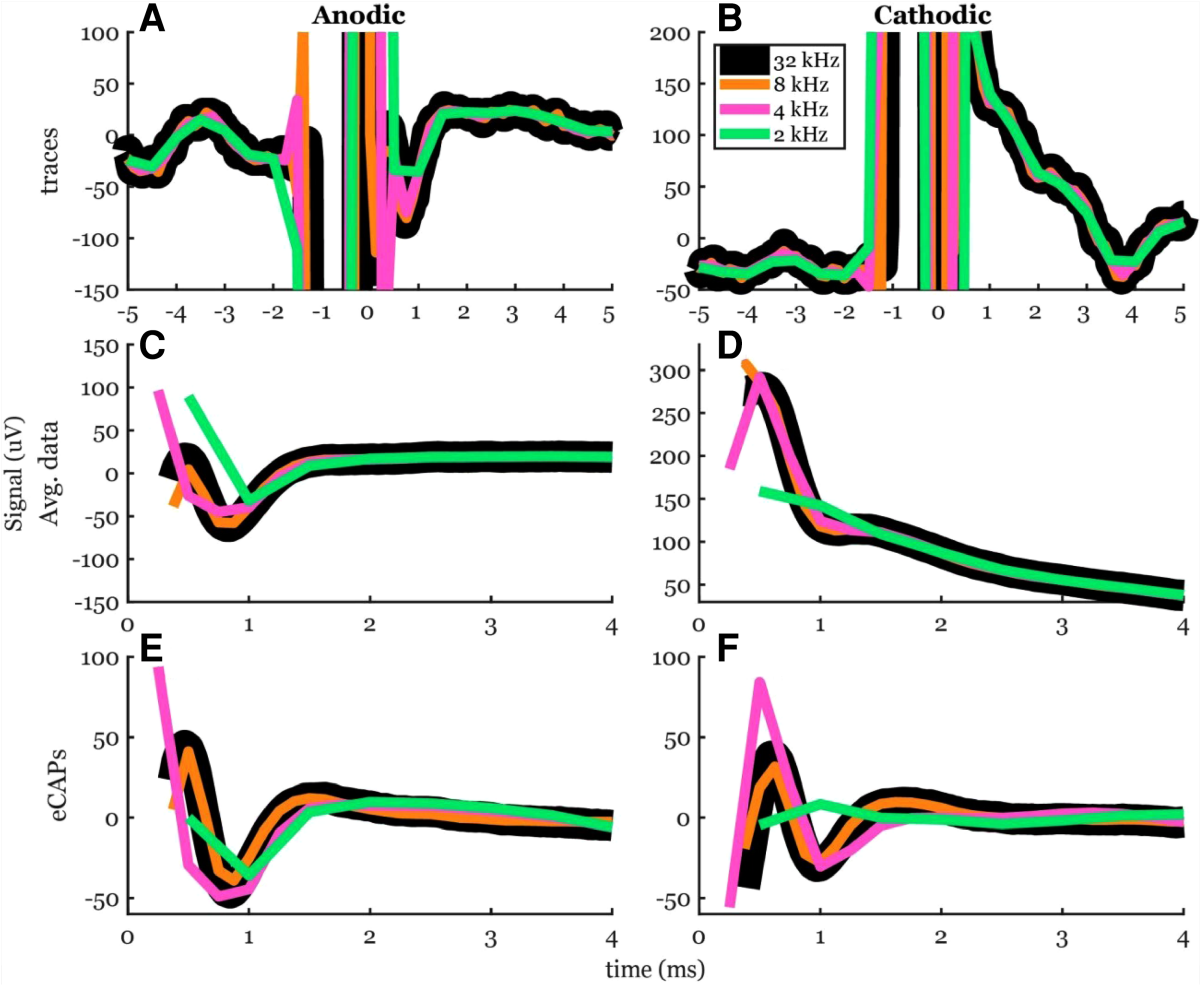
Effect of sampling rate on ECAP morphology. (**A,B**) Example time traces at the original 32 kHz and the down-sampled 8, 4, and 2 kHz with an offline antialiasing filter. (**C,D**) The traces’ average within the fit window for stim artifact removal. (**E,F**) The resulting anodic and cathodic ECAPS respectively.

We tested lower sampling rates of 8, 4, and 2 kHz and compared the resulting ECAP metrics to the 32-kHz case (Figure 9). The ECAP morphology became distorted when sampling was lower than 8 kHz. At 8 kHz, a rough ECAP morphology can be detected. At 4 kHz, the morphology may be identified, but the ECAP metrics were poorly captured.

**Figure 10:**
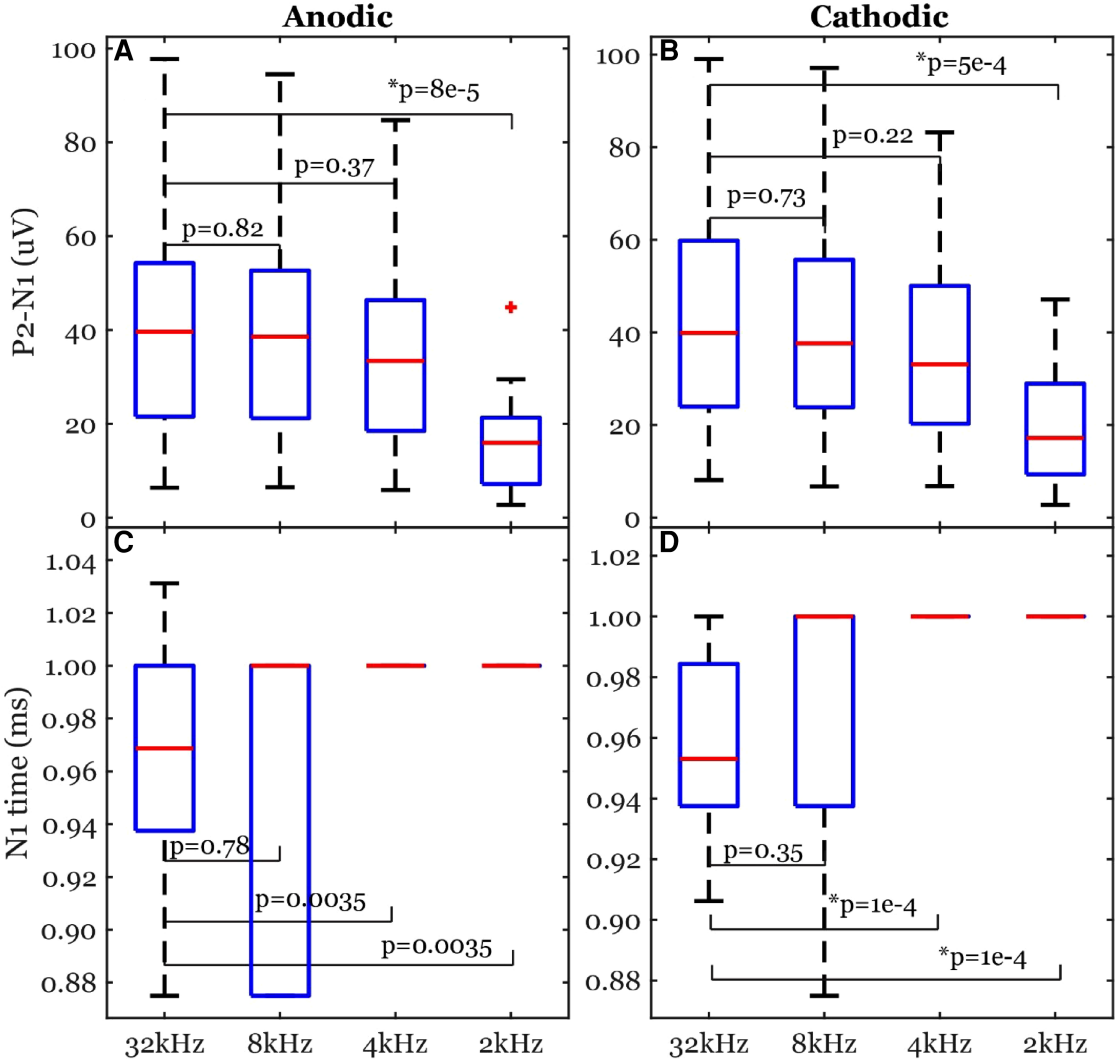
Common ECAP characteristics: (**A,B**) peak-valley amplitude (P2-N1) and (**C,D**) N1 time; across the four sampling frequencies: 32, 8, 4, and 2 kHz. The *p*-values are established from linear models. The data included 20 observations from two participants with stimulation amplitudes sufficient to evoke ECAP in each participant.

Peak-valley amplitude (P2-N1) was affected by the sampling rate (Figure 10). The algorithm detected peaks for all cases of sampling frequencies. The peak-valley amplitude was not significantly different between 32 kHz and each of 8 and 4 kHz (Figure 10). The 2-kHz's peak-valley amplitude was significantly different from the 32-kHz case in both anodic and cathodic ECAP. The average peak-valley differences of 8, 4, and 2 kHz from 32  kHz were 1.4, 5.5, and 25.3 µV in anodic ECAP, and 2.3, 8.3, and 24.3 µV in cathodic ECAP, respectively.

The N1 times were affected by the sampling rate as well (Figure 10). The algorithm detected N1 times for all cases. The N1 times were significantly different between 32 kHz and each of 4 and 2 kHz in the cathodic ECAPs. The average N1-time differences of 8, 4, and 2 kHz from 32 kHz were 3, −34, and −34 µs in anodic ECAP, and −9, −41, and −41 µs in cathodic ECAP, respectively. Given that our time resolution is 31.25 µs (1/32 kHz), the average difference of N1-time in 8 kHz from 32 kHz is indistinguishable from zero.

#### 4.2.2. Online filter

**Figure 11:**
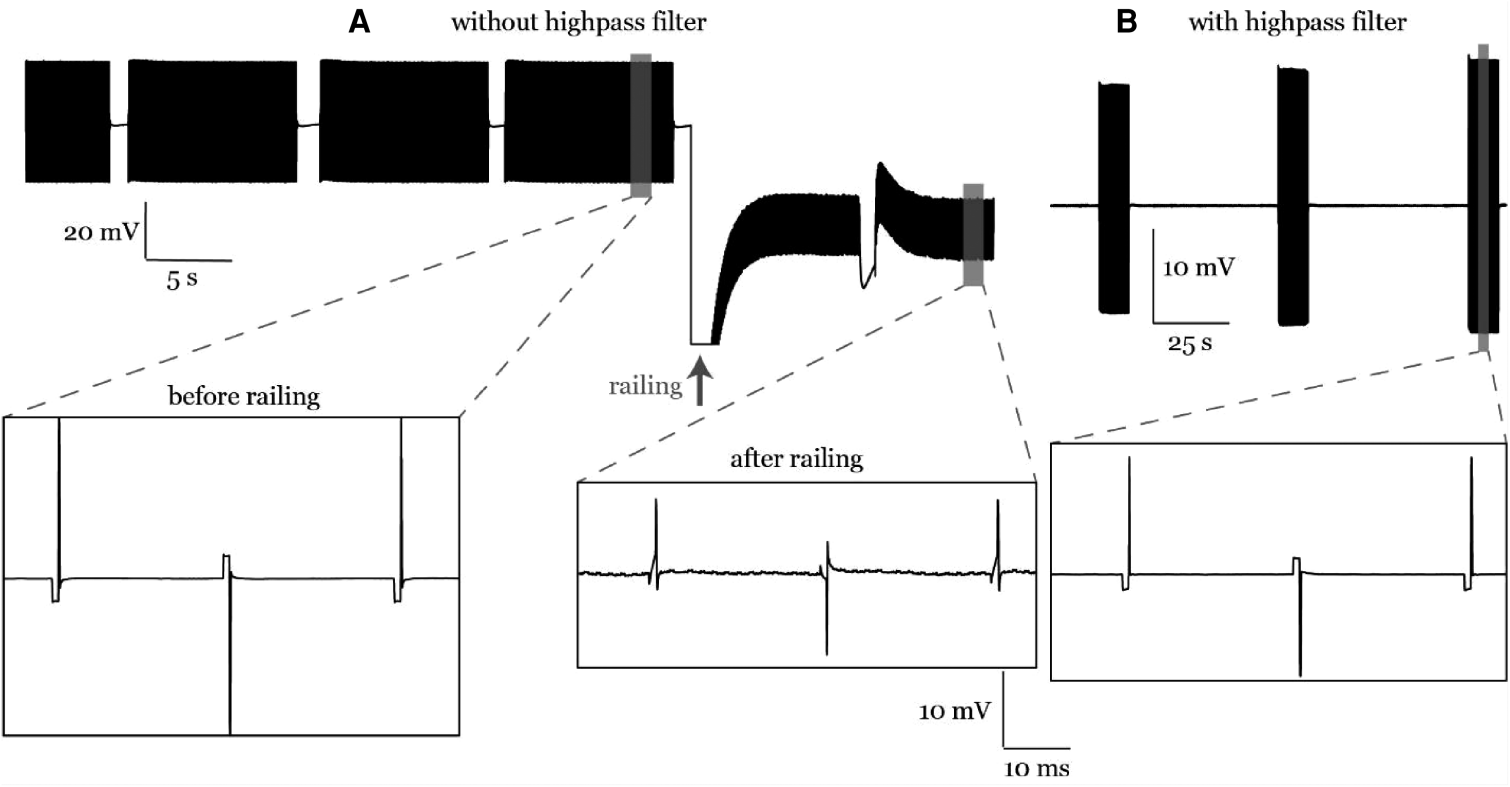
(**A**) A railing case in participant #1. The stimulation artifact curve followed the stimulation waveform before railing and was distorted after railing. (**B**) Railing was eliminated later in participant #2 using an online high pass filter during recording.

Without the ATLAS pre-emphasis (high pass) filter, the recordings may saturate the input range on the continuous files (.ncs, 16-bit). In participant #1 the signal drifted and hit the rails during the stimulation artifact (Figure 11) and subsequently, the shape of the stimulation artifact changed dramatically becoming much more triangular pulse than the true rectangular waveform. However, the change in the waveform was seen in the 16-bit data format but was preserved in the 24-bit data format. To be cautious and prevent it from happening again in subsequent participants, a 0.1-Hz high pass filter in the ATLAS system was enabled to keep the signal centered around zero and prevent railing. This change in the waveform following the railing event was unexplained, and we provide this as a cautionary tale. This railing incident occurred in one experiment during visit 3 of participant 1. One experiment (recording), out of many collected on that visit, was affected by railing and was not included in the ECAP analysis presented here.

## 5. Discussion

We investigated some key variations in hardware configurations, artifact removal methods, and filtering options to quantify the metrics of ECAP component in SCS. Overall, our results indicate that for a reliable ECAP characterization, a sampling rate of at least 8 kHz is required together with an online high pass filter having a very low cutoff frequency (e.g., 0.1 Hz). Using on-lead or patch-on-back ground, in one participant each, did not affect our ability to detect ECAPs. In addition, subtracting a double exponential fit from the raw recording can eliminate the artifact and preserve the ECAP morphology. A median filter may be applied before fitting if the raw data contains baseline drift.

### 5.1. Lead placements

In this study, stimulation was applied through the most caudal two channels, and the two implanted leads had an offset of 1–2 electrodes (Figure 1). To minimize the effect of stimulation artifact in the recorded evoked responses, the biggest possible offset along the cephalad-caudal axis between the two implanted leads should be maintained, which indicates that the lead length is often the limiting factor of recording ECAPs (16). However, the leads placement in clinical practice often used two leads placed in parallel with at most a 0–3 contact offset (7). Our study demonstrated the feasibility of using one of the clinical practices with a 1–2 contact offset from two implanted leads to record evoked responses (Figure 1). In this setup, electrodes close to the stimulation and reference sites were distorted with significant artifacts (data not shown).

Our reference electrode was on-lead (#9). For ground, we used a patch on the back and electrode #16 in participants #1 and #2, respectively. ECAPs were detected in both cases. Power spectrum analysis of a single channel during no stimulation showed relatively larger 60-Hz noise peak compared to the surrounding frequency baseline in the patch-on-back compared to the on-lead (data not shown). These two schemes are the only feasible pain-free options in externalized human studies. Local tissue reference (implanting another lead or plate into a nearby muscle tissue) is common in animal studies (19), but not feasible in human studies.

### 5.2. Hardware and filtering settings

From this study, the lowest recommended sampling rate to record ECAP signal without distortion is 8 kHz. Most investigational devices exceed this limit (Table 1). Some FDA-approved clinical devices also exceed this limit (e.g., Natus Quantum™ Amplifier with 16 kHz). In this study, recording sampling rate is at 32 kHz, which was different from the stimulation sampling rate (40 kHz). Therefore, the exact zero time of the stimulation onset with respect to the sampling interval varied, resulting in a slow beating phenomenon of the stimulation artifacts (data not shown). For optimal stimulation artifact removal, it is best to match the recording and stimulation sampling frequencies to phase-lock the zero-time events.

Different stimulation configurations have been used in previous research, such as bipolar and tripolar stimulation (Table 1). These spatial stimulation configurations were assessed in simulation studies (20–23), *in vivo* in animals and humans (16). The tripolar configuration significantly reduces the stimulation artifact compared to the bipolar configuration when using symmetrical biphasic waveforms. The downside of a tripolar configuration is a higher threshold to evoke ECAP, which means faster battery discharge for implanted devices (16). In this study, we select a bipolar configuration with asymmetric biphasic waveform (Figure 3). It helps to reduce the stimulation artifact overlapping with evoked potentials.

We also investigated the effect of different offline filters on the ECAP signals. The offline filters of Med and HP yielded similar ECAP metrics (peak-valley amplitude and N1 time) to that of the raw data (Figure 8). On the other hand, the LP filter introduced ringing effect close to the stimulation pulse (Figure 7). Thus, it can cause distortion of the recorded ECAPs in peak timings and magnitudes. This can be attributed to the low cutoff frequency (3 kHz) compared to the ECAP Nyquist frequency (≥2 kHz). Using a higher cutoff frequency of 7.5 kHz could improve the ECAP metrics (data not shown). For offline processing, we recommend using the raw data, and if measurement noise becomes an issue, then the median filter would be recommended. In addition, most previous studies did not report their online filters if any (Table 1). Eliminating online filters would be helpful to eliminate ripples following the stimulation artifact in the filtered signal. However, our study indicates an online high pass filter with a very low cutoff frequency (e.g., 0.1 Hz) may be required to avoid railing (Figure 11).

### 5.3. Different strategies to remove the stimulation artifact

A major challenge in processing the evoked responses recorded by SCS lead is to remove the stimulation artifact. Here, different forms of curve-fitting-based artifact removal methods were investigated. From our results, the best artifact curve fitting approach to remove artifact was found to be a double exponential model (Figure 5), which suggests that the tissue impedance and the electrode impedance are not identical, and a second-order dynamical system model is better suited to fit the superposition of their two decays (24–26). Results shown here were obtained using a double exponential curve fit subtracted from the raw recording. Similar decay morphologies have been observed in the same participant due to different stimulation amplitudes and polarities (Figures 4, 6). However, this may not always be the case. Our results are consistent with previous reports where researchers used three artifact removal methods: a curve fit (exponential + ramp), a differentiator, and correlation to generate a template (15). Their exponential-plus-ramp curve may approximate our double exponential in a shorter temporal window (ours was 3.625 ms vs. their 1.5 ms). Moreover, their curve fit method outperformed the other methods. Taken together, the curve-fit-based artifact removal methods are strongly recommended.

The second-best curve fit that can remove the stimulation artifact is a second order polynomial: poly2 (see R^2^ in Figure 5). The major advantage of poly2 over exp1 and exp2 is the significantly shorter computation time (Figure 5). Solving linear least squares problems in poly2 is much faster and can be done analytically. In addition, the nonlinear least squares algorithms for exp1 and exp2 are iterative and depends on the selection of a starting point, which means it can get stuck in a local minimum. Interestingly, the N1 times and peak-valley amplitudes are similar between poly2 and exp2. Therefore, poly2 is likely to be a better option for a real-time closed-loop applications in embedded systems. One thing worth noting is that all artifact removal methods, including curve-fit-based methods, may overestimate or underestimate the neural response (15). Nevertheless, exp2 and poly2 curves cannot produce a triphasic morphology (similar to P1-N1-P2), so they would not artificially introduce ECAP-like signals when there is none.

Since the artifact is related to the passive properties (impedance) of the electrical interface between the electrodes and the tissue, the artifact response is supposed to be linear with the stimulation amplitude. Therefore, the artifact trends are a useful visual tool to tell whether the curve-fit estimation was accurate. When plotting the best fit curves of artifacts normalized by the stimulation amplitudes for a given participant, one expects the curves to match and coincide. Different artifact trends (flat, initially negative increasing towards zero, and initially positive decreasing towards zero) were observed between participants (15). We observed similar trends in the same participant (Figures 4, 6) due to different stimulation polarities (anodic vs. cathodic) and different curve fits. For example, exp1 fit in Figure 4C shows a flat trend compared to the initially negative and increasing towards zero trend in exp2 and poly2.

In addition, a previous study has also shown that processing the evoked responses induced by opposite stimulation polarities may be used to eliminate stimulation artifact (16). In this study, we also investigate this by switching polarity of stimulation using two most caudal electrodes. From our results, different N1 latencies were observed when triggered by anodic and cathodic stimulation (Figure 5). Overall, the anodic ECAP's N1 latency is sooner than the cathodic ECAP's N1 latency by 164 µs on average using the exp2 fit. This phase shift is attributed to the spatial offset in the stimulation sites when using anodic and cathodic pulses (16). Moreover, the anodic ECAP had a larger amplitude (P2-N1) than the cathodic ECAP by 32.48 µV on average (Figures 4, 5). For these two reasons, averaging anodic and cathodic pulses together to eliminate stimulation artifacts will distort some characteristics of ECAPs. In short, the signal obtained using two different stimulation polarities between two contacts should be analyzed separately.

## 6. Conclusion

**Figure 12:**
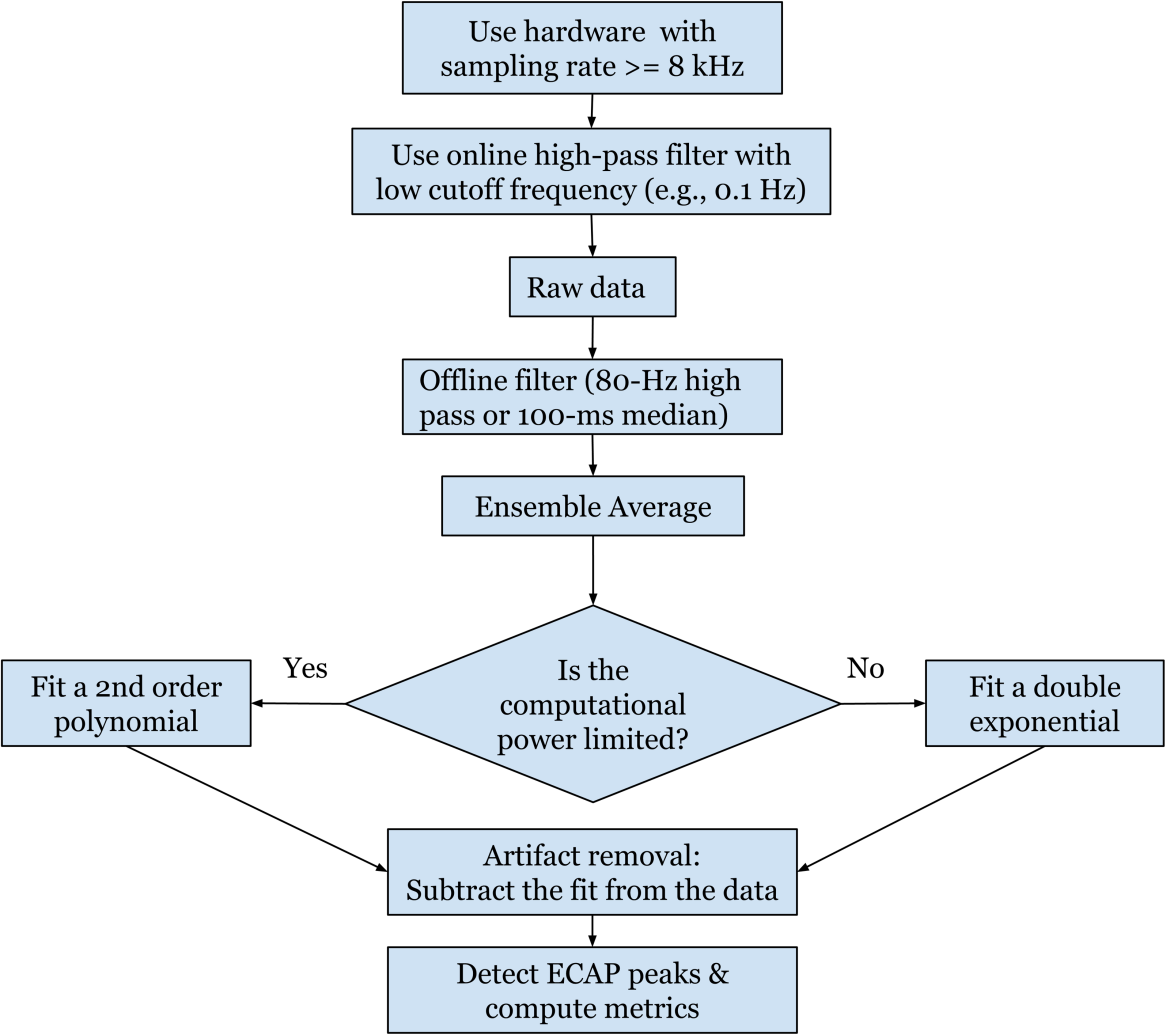
A flowchart summarizing the recommended settings for a robust and standardized protocol for measuring ECAPs.

In conclusion, this study determines the methods and system specifications found to be useful for recording and processing ECAP components. In short, ordinary lead configuration in clinical practice with a 1–2 contact offset could enable ECAP signal recording when using most caudal two contacts for stimulation and cephalad contacts for recording. The lowest sampling rate for non-distorted evoked response recording is around 8 kHz. Flipping polarity of stimulation contacts might help to reveal additional evoked response characteristics, and thus averaged signals recorded from the two flipped polarities will cause information loss and thus is not recommended. Subtracting the poly2 based curve fitting from the raw recording could remove residual stimulation artifact and presents a feasible application in embedded systems because of low computational expenses. A summary of these findings is represented in a flowchart in Figure 12.

The results shown here are from findings based on a small number of participants and not an extensive study. Data from more patients should be collected to account for the variability across different participants. In addition, online signal processing should be investigated more to understand potential challenges in applications on an embedded system.

## Data Availability

The data used in this study was fully funded by Abbott and is the proprietary and confidential property of Abbott. Abbott is under no obligation to release the data to third parties.
